# Porous Zinc Oxide and Plant Polyphenols as a Replacement for High-Dose Zinc Oxide on Growth Performance, Diarrhea Incidence, Intestinal Morphology and Microbial Diversity of Weaned Piglets

**DOI:** 10.3390/ani14030523

**Published:** 2024-02-05

**Authors:** Dongxu Ming, Jizhe Wang, Chenggang Yin, Yiqun Chen, Yanpin Li, Wenjuan Sun, Yu Pi, Alessandra Monteiro, Xilong Li, Xianren Jiang

**Affiliations:** 1 Key Laboratory of Feed Biotechnology of Ministry of Agriculture and Rural Affairs, Institute of Feed Research, Chinese Academy of Agricultural Sciences, Beijing 100081, China; mdx920825@163.com (D.M.); b20223040396@cau.edu.cn (J.W.); ycg0701@126.com (C.Y.); liyanpin@caas.cn (Y.L.); sunwenjuan@caas.cn (W.S.); piyu@caas.cn (Y.P.); 2 Key Laboratory of Feed Synthetic Biotechnology of Ministry of Agriculture and Rural Affairs, Ganzhou 341000, China; 3 State Key Laboratory of Animal Nutrition, College of Animal Science and Technology, China Agricultural University, Beijing 100193, China; 4 Sanya Institute of China Agricultural University, Sanya 572000, China; 5 Animine, 74960 Annecy, France; ychen@animine.eu (Y.C.); amonteiro@animine.eu (A.M.)

**Keywords:** growth performance, intestinal health, plant polyphenols, porous zinc oxide, weaned piglets

## Abstract

**Simple Summary:**

The addition of high doses of zinc oxide to the diet of weaned piglets can effectively promote their growth performance and reduce diarrhea. However, the zinc oxide added to the piglet diet has a higher concentration, and a considerable part of it is excreted from the body through urine and feces, resulting in serious environmental pollution. Porous zinc oxide and plant polyphenols have garnered significant attention in recent years due to their potential as novel feed additives. This study investigated the effects of porous zinc oxide and plant polyphenols on the growth performance, diarrhea incidence, and intestinal health of weaned piglets to evaluate whether porous zinc oxide and plant polyphenols have similar effects to high-dose zinc oxide in improving the intestinal health of piglets.

**Abstract:**

The aim of this experiment is to evaluate the effects of adding porous zinc oxide, plant polyphenols, and their combination to diets without antibiotics and high-dose zinc oxide on the growth performance, diarrhea incidence, intestinal morphology, and microbial diversity of weaned piglets. A total of 150 Duroc × Landrace × Large White weaned piglets were allocated to one of five diets in a randomized complete block design with six replicates and five piglets per replicate. The experimental period was 42 d, divided into two feeding stages: pre-starter (0–14 d) and starter (14–42 d). In the pre-starter stage, the negative control group (NC) was fed a basal diet, the positive control group (PC) was fed a basal diet with 2000 mg/kg of zinc oxide, the porous zinc oxide group (PZ) was fed a basal diet with 500 mg/kg of porous zinc oxide, the plant polyphenol group (PP) was fed a basal diet with 1500 mg/kg of plant polyphenols, and the combination group (PZ + PP) was fed a basal diet with 500 mg/kg of porous zinc oxide and 1500 mg/kg of plant polyphenols. In the starter stage, the NC, PC, and PZ groups were fed a basal diet, while the PP and PZ + PP groups were fed a basal diet with 1000 mg/kg of plant polyphenols. The results showed that, (1) compared with the PZ group, adding plant polyphenols to the diet showed a trend of increasing the ADFI of weaned piglets from 14 to 28 d (*p* = 0.099). From days 28 to 42 and days 0 to 42, porous zinc oxide and the combination of porous zinc oxide and plant polyphenols added to the control diet improved the FCR to the level observed in pigs fed the PC diet. (2) Dietary PZ + PP tended to increase the jejunal villus height (VH) of weaned piglets (*p* = 0.055), and significantly increased the villus-height-to-crypt-depth ratio compared to the NC group (*p* < 0.05). (3) Compared with the NC group, PZ supplementation decreased the relative abundance of Firmicutes and increased the relative abundance of Bacteroidetes, and the relative abundance of *Lactobacillus* in the PZ and PZ + PP groups were both increased. In conclusion, porous zinc oxide and plant polyphenols may have synergistic effects in modulating intestinal health in weaned piglets and be a potential alternative to high-dose zinc oxide.

## 1. Introduction

Diarrhea in weaned piglets is a significant issue in the pig industry, often accompanied by weaning stress. These problems can lead to decreased growth performance, increased mortality rates, and enormous economic losses to production [1]. Antibiotics have been widely used as feed additives to prevent and treat weaned piglets’ diarrhea. However, the overuse of antibiotics has led to increased antibiotic resistance, posing a severe threat to public health. Many studies have found that zinc oxide has biological functions such as reducing diarrhea [2,3], improving growth performance [4,5], improving intestinal morphology [6,7], and improving the structure of intestinal microbiota [8,9]. Adding high-dose zinc oxide to their diet can improve the growth performance of weaned piglets by alleviating weaning diarrhea and promoting healthy intestinal development, effectively reducing the need for antibiotics. However, the long-term feeding of high-dose zinc oxide diets can lead to a waste of zinc sources [10], the inhibition of absorption of other minerals [11], and zinc poisoning [12,13]. The European Union banned the use of high-dose zinc oxide in feed in June 2022. Therefore, finding an efficient and green new type of feed additive is very important for livestock production.

In recent years, porous zinc oxide and plant polyphenols have garnered significant attention due to their beneficial effects on the growth and gut health of weaned piglets. Porous zinc oxide, a novel zinc oxide derivative, is composed of aggregated particles with diameters less than 1 μm, resulting from the interconnection of nanoscale zinc oxide needle-like crystals. This material boasts a substantial specific surface area, excellent fluidity, and high mixing uniformity. It can effectively increase the contact area with intestinal chyme and pathogenic bacteria, thereby fostering a more favorable environment for the biological function of zinc oxide [13]. Plant polyphenols are abundant in various plant tissues such as stems, leaves, and fruits. They possess a range of biological activities, including anti-inflammatory [14,15,16], antibacterial [17], antioxidant [18,19], and improving intestinal permeability [20,21]. Currently, research is primarily focused on organic zinc (e.g., protein-bound zinc, amino acid chelated zinc), inorganic zinc (e.g., zinc sulfate, basic zinc chloride), and advanced zinc oxide products like coated zinc oxide and nano zinc oxide. However, the utilization of porous zinc oxide in weaned piglets remains in its early stages, with insufficient explorations of its potential synergistic effects when combined with plant polyphenols. The aim of this experiment was to explore the regulatory effects and mechanisms of porous zinc oxide, plant polyphenols, and their complexes on the growth performance and intestinal health of weaned piglets, and to provide the basis for porous zinc oxide and plant polyphenols to replace high-dose zinc oxide in pig production.

## 2. Materials and Methods

This study was approved by the Animal Care and Use Committee of the Feed Research Institute of the Chinese Academy of Agricultural Sciences.

### 2.1. Piglets and Experimental Design

A total of 150 Duroc × Landrace × Large White crossbred weaned barrows (28 ± 2 days of age; BW = 7.19 ± 1.27 kg) were allocated to one of five diets in a randomized complete block design with six replicates and five piglets per replicate. The experimental period was 42 d, divided into two feeding stages: pre-starter (0–14 days) and starter (14–42 days). In the pre-starter phase, the negative control group (NC) was fed a basal diet, the positive control group (PC) was fed a basal diet with 2000 mg/kg of zinc oxide, the porous zinc oxide group (PZ) was fed a basal diet with 500 mg/kg of porous zinc oxide, the plant polyphenol group (PP) was fed a basal diet with 1500 mg/kg of plant polyphenols, and the combination group (PZ + PP) was fed a basal diet with 500 mg/kg of porous zinc oxide and 1500 mg/kg of plant polyphenols. In the starter phase, the NC, PC, and PZ treatment groups were fed the basal diet, while the PP and PZ + PP groups were fed the basal diet with 1000 mg/kg of plant polyphenols. The plant polyphenols mainly contained lignin polyphenols (tannins) that were purchased from Methodo Chemicals S.r.l., Italy. The based diet was formulated to meet the National Research Council (2012) recommendations for weaned piglets [22]. The composition and nutritional level of the experimental basal diet are shown in Table 1.

### 2.2. Sample Collection and Processing

On day 14 of the experiment, one pig was selected from each replicate and slaughtered using an electric shock. After slaughter, the duodenum, jejunum, ileum, and cecum were dissected immediately. Tissue samples (about 1 to 2 cm) were taken from the duodenum, jejunum, and ileum, fixed with a 4% formaldehyde–phosphate buffer, and kept at 4 °C for a microscopic evaluation of the mucosal morphology. The cecal contents were quickly frozen in liquid nitrogen and the sample was then stored at −80 °C.

### 2.3. Growth Performance and Diarrhea Incidence Measurement

The body weight was measured on days 0, 14, 28, and 42, and feed disappearance and feed intake on these days was recorded. The average daily gain (ADG), average daily feed intake (ADFI), and feed conversion (F:G) were calculated based on weight and feed measurements. Diarrhea scores were recorded daily for all piglets from days 0 to 14 by the same person and were based on the following scale: 1 = hard, dry pellet; 2 = firm, formed stool; 3 = soft, moist stool that retains its shape; 4 = soft, unformed stool; and 5 = watery liquid that can be poured. A liquid consistency (score 4–5) was considered indicative of diarrhea [23]. The incidence of diarrhea for weaned piglets in each pen was calculated as [(number of weaned piglets with diarrhea × number of days of diarrhea)/(total number of weaned piglets × number of days of the experiment)] × 100 [24].

### 2.4. Intestinal Morphology

The samples of the duodenum, jejunum, and ileum were removed from the fixed solution and treated with water flushing, gradient alcohol dehydration, xylene transparency, and paraffin embedding. Sections were taken at a thickness of 4 μm and stained with hematoxylin–eosin (HE). The data of villus height (VH) and crypt depth (CD) were measured using an Image Pro-Plus 6.0 Software Analysis System (Media Cybernetics, Singapore). Six fields were randomly selected to read the VH and CD, and the ratio of villus height to crypt depth (VCR) was calculated.

### 2.5. Microbiology of Cecal Contents

The total DNA of the sample microbial community was extracted, and the quality of the extracted DNA was assessed via 1% agarose gel electrophoresis to determine its concentration and purity. Subsequently, primers were synthesized based on the determined region and PCR amplification was performed on the highly variable region (V3–V4) of the 16S rDNA gene. The PCR products from the same sample were pooled, and a 2% agarose gel was prepared for purification. The purified DNA was detected and quantified, and a library was constructed, followed by amplicon sequencing using Miseq. Post-sequencing, splicing, quality control, and de-splicing were performed on the original sequencing data to obtain an optimized sequence. This optimized sequence was subjected to operational taxonomic unit (OTU) clustering, chimeric elimination, and taxonomic annotation (utilizing the silva138/16s bacteria species classification database with a classification confidence level of 0.7) based on a similarity level threshold of 97% to facilitate subsequent species diversity analysis.

### 2.6. Statistical Analysis

All experimental data except for the diarrhea incidence were analyzed using the GLM Procedure of SAS as a randomized complete block design (SAS Inst. Inc., Cary, NC, USA). Differences in diarrhea incidence among treatments were tested by the procedure GLIMMIX. Differences among means were evaluated by the Student–Newman–Keuls test. Treatment effects were significant if *p* ≤ 0.05. When 0.05 < *p* ≤ 0.10, it was considered to have a trend.

## 3. Results

### 3.1. Growth Performance and Diarrhea Incidence

The results of PZ, PP, and PZ + PP supplementation on the growth performance and diarrhea incidence of weaned piglets are shown in Table 2. The addition of porous zinc oxide and plant polyphenols alone or in combination did not significantly affect the body weight and ADG of the weaned piglets. Compared with the PZ group, adding plant polyphenols to the diet showed a trend of increasing the ADFI of the weaned piglets from 14 to 28 days (*p* = 0.099). From days 28 to 42 and days 0 to 42, porous zinc oxide and the combination of porous zinc oxide and plant polyphenols added to the control diet improved the FCR to the level observed in pigs fed the PC diet, while the addition of plant polyphenols increased the FCR (*p* < 0.05). And each treatment group had no significant effect on the diarrhea incidence of the weaned piglets (*p* > 0.05).

### 3.2. Intestinal Morphology

The effects of adding porous zinc oxide and plant polyphenols to the diet on the intestinal morphology of the weaned piglets are shown in Table 3. Compared with the NC group, adding plant polyphenols to the diet showed a trend of increasing the duodenal VH in the weaned piglets (*p* = 0.077), but had no significant effect on the CD and VCR. Dietary high-dose ZnO and PZ + PP tended to increase the jejunal VH of the weaned piglets (*p* = 0.055), while significantly increasing the VCR compared to the NC group (*p* < 0.05). There was no significant dietary effect on the VH and CD of the ileum (*p* > 0.05), while piglets in the PC group showed an increasing trend in the VCR compared to the NC group (*p* = 0.052).

### 3.3. Microbiolota in Cecal Contents

No significant difference was observed in terms of α diversity in the cecal contents of weaned piglets among all the dietary treatments (Table 4, *p* > 0.05).

**Figure 1:**
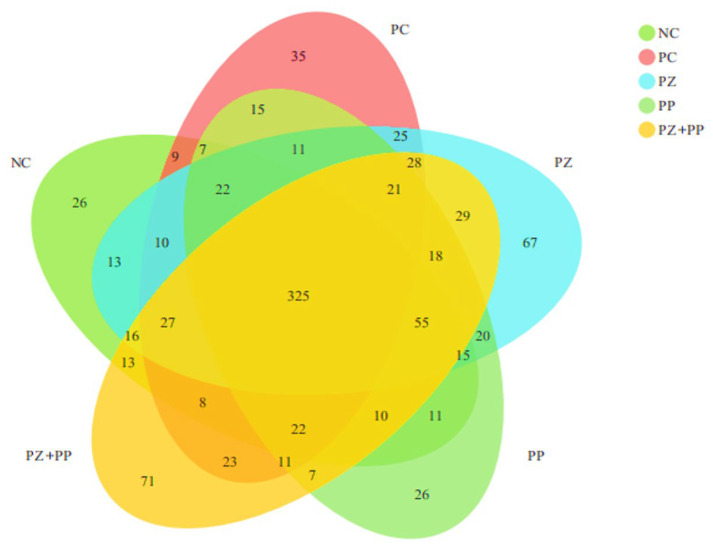
Effect of porous zinc oxide (PZ), plant polyphenols (PP), or their combination (PZ + PP) supplementation on bacterial diversities in the cecum content of weaned piglets compared to a basal diet and the basal diet containing ZnO. NC = basal diet; PC = basal diet with 2000 mg/kg of zinc oxide in the pre-starter phase; PZ = basal diet with 500 mg/kg of porous zinc oxide in the pre-starter phase; PP = basal diet with 1500 mg/kg of plant polyphenols in the pre-starter phase and 1000 mg/kg of plant polyphenols in the starter phase; PZ + PP = basal diet with 500 mg/kg of porous zinc oxide and 1500 mg/kg of plant polyphenols in the pre-starter phase and 1000 mg/kg of plant polyphenols in the starter phase.

There are 325 OTUs in the NC, PC, PZ, and PZ + PP groups (Figure 1). The unique OTUs in the NC, PC, and PP groups are 26, 35, and 26, respectively. The PZ and PZ + PP groups have a higher number of unique OTUs at 67 and 71, respectively.

**Figure 2:**
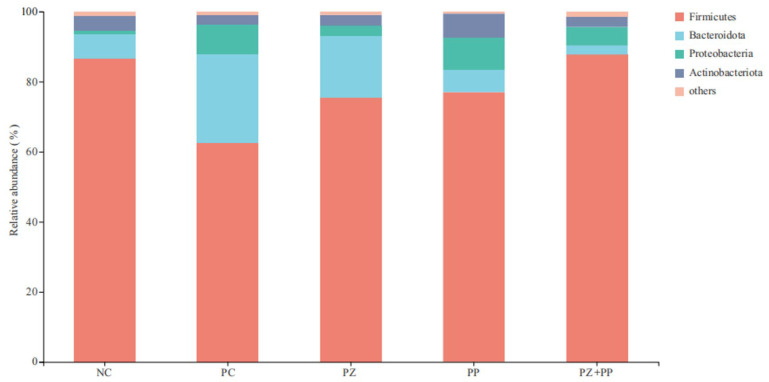
Analysis diagram of the community composition in the cecum content of weaned piglets at the phylum level. NC = basal diet; PC = basal diet with 2000 mg/kg of zinc oxide in the pre-starter phase; PZ = basal diet with 500 mg/kg of porous zinc oxide in the pre-starter phase; PP = basal diet with 1500 mg/kg of plant polyphenols in the pre-starter phase and 1000 mg/kg of plant polyphenols in the starter phase; PZ + PP = basal diet with 500 mg/kg of porous zinc oxide and 1500 mg/kg of plant polyphenols in the pre-starter phase and 1000 mg/kg of plant polyphenols in the starter phase.

After conducting a clustering analysis of the total reads based on sequences with an identity ≥ 97%, Firmicutes, Bacteroidetes, Proteobacteria, and Actinobacteria are the dominant microbiota at the level of the cecal microbiota community (Figure 2). The relative abundance of Firmicutes in the NC, PC, PZ, PP, and PZ + PP groups was 86.56%, 62.65%, 75.39%, 77.01%, and 87.75%, respectively. The relative abundance of Bacteroidetes in the NC, PC, PZ, PP, and PZ + PP groups was 7.06%, 25.15%, 17.74, 6.35%, and 2.58%, respectively. The relative abundance of Proteobacteria in the NC, PC, PZ, PP, and PZ + PP groups was 1.04%, 8.59%, 2.83%, 9.17%, and 5.30%, respectively.

**Figure 3:**
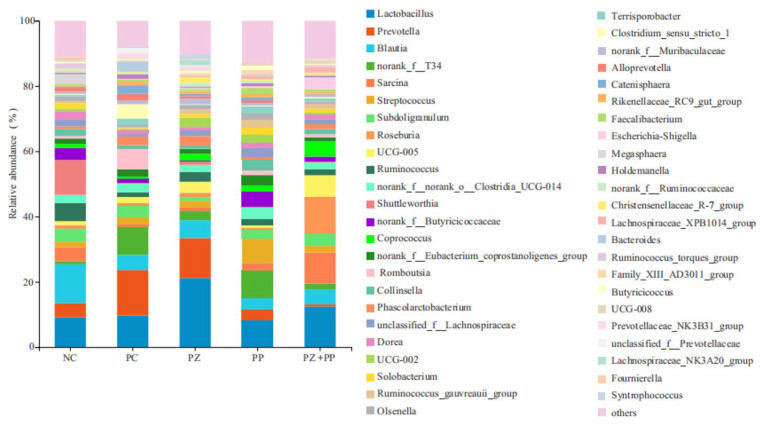
Analysis diagram of the community composition in the cecum content of weaned piglets at the genus level. NC = basal diet; PC = basal diet with 2000 mg/kg of zinc oxide in the pre-starter phase; PZ = basal diet with 500 mg/kg of porous zinc oxide in the pre-starter phase; PP = basal diet with 1500 mg/kg of plant polyphenols in the pre-starter phase and 1000 mg/kg of plant polyphenols in the starter phase; PZ + PP = basal diet with 500 mg/kg of porous zinc oxide and 1500 mg/kg of plant polyphenols in the pre-starter phase and 1000 mg/kg of plant polyphenols in the starter phase.

From Figure 3, at the genus level, the community microorganisms are mainly concentrated in *Lactobacillus*, *Prevotella*, *Blautia*, *norank_f_T34*, and *Sarcina*. The relative abundance of *Lactobacillus* in NC, PC, PZ, PP, and PZ + PP groups was 9.12%, 9.71%, 21.19%, 8.40%, and 12.44%, respectively. The relative abundance of *Prevotella* in the NC, PC, PZ, PP, and PZ + PP groups was 4.32%, 13.95%, 12.23%, 3.16%, and 0.66%, respectively. The relative abundance of *Blautia* in the PC, PZ, PP, and PZ + PP groups decreased by 6.44–8.46% compared to the NC group. The relative abundance of *Sarcina* in the PC group, PZ group, and PP group decreased compared to the control group, while the relative abundance in the PZ + PP group increased.

## 4. Discussion

It is well understood that adding zinc oxide to the diet can reduce the diarrhea incidence of weaned piglets, but the effect on improving growth performance is inconsistent. Adding high-dose zinc oxide (3000 mg/kg) or porous zinc oxide at a lower level (750 or 1500 mg/kg) to the diet showed a significant increase in the ADG of weaned piglets, and significantly decreased the FCR and diarrhea incidence [25]. A previous study found that high-dose zinc oxide and intermediate doses of porous zinc oxide (500 mg/kg) could significantly increase the ADFI of weaned piglets from 0 to 28 d and significantly reduce their diarrhea rates [13]. The improvement in growth performance when porous zinc oxide is supplemented can be related to the higher specific surface area in comparison to standard sources of ZnO [26]. This leads to a better bioavailability [27], better bacteria control [28], and improved performance, even at lower dosages [29]. Some studies also found that the addition of high-dose zinc oxide had no significant effect on the body weight of weaned piglets on day 14, as well as the ADG, ADFI, and FCR during days 0–14 [30]. In this study, it was found that the diarrhea incidence in the PC, PZ, PP, and PZ + PP groups was not statistically significant; this might be due to the sound growth condition of the pigs and the lack of pathogenic stressors. In terms of growth performance, the BW, ADG, ADFI, and FCR of the weaned piglets in the PC and PZ groups were not significantly different from those in the NC group. Compared with the NC group, the FCR of the weaned piglets in the PC group and PZ group decreased by 10.71% and 9.52% from days 0 to 14. From days 14 to 28, the FCR of the PC and PZ groups was 5.84% and 6.83% lower than the NC group, which indicated that high-dose zinc oxide and porous zinc oxide had potential in improving the growth performance of the weaned piglets. There was no significant difference in FCR between the NC, PC, and PZ groups from days 28 to 42, indicating that the continuity of high-dose zinc oxide and porous zinc oxide (500 mg/kg) in improving growth performance was mainly concentrated in the first two weeks after the dietary supplementation of the two, and then the effect gradually decreased. It has been found that supplementation of Eucommia flavone in a low-protein diet can significantly improve the ADG and FCR of weaned piglets from 15 to 35 days and 0 to 35 days, and significantly reduces the diarrhea incidence of piglets during 0–15 and 0–35 d [31]. In this study, we observed that plant polyphenol supplementation increased the FCR compared to the NC group, which may suggest that the absorption of lignin polyphenols is not complete, thus affecting its effect. However, the growth performance and diarrhea incidence of the PZ + PP piglets had no difference from piglets in the PC group, indicating that porous zinc oxide and plant polyphenols may have a synergistic effect in improving the growth performance of weaned piglets, and dietary porous zinc oxide might compensate for the deficiency of plant polyphenols.

The intestine is the main organ for nutrient absorption, and the VH and CD of small-intestinal epithelial cells and their ratio are important indicators to measure intestinal morphology. Research has found that the VCR of the duodenum, jejunum, and ileum of weaned piglets can be significantly increased by dietary high-dose zinc oxide [32]. Previous studies demonstrated that enterotoxigenic *Escherichia coli* K88 infection could lead to a decrease in the VH and a deepening of the CD. And dietary additions of high-dose zinc oxide can significantly increase the VCR of the jejunum and ileum of weaned piglets after challenge [33]. In this study, dietary supplementation with high-dose zinc oxide improved some aspects of morphology in the ileum and jejunum of weaned piglets, and plant polyphenols improved the duodenal VH. Previous studies demonstrated that resveratrol significantly increased the jejunal VH and VCR of piglets and significantly increased the jejunal mucosa ZO-1 mRNA expression level [34]. Dietary supplementation of 30 or 90 mg/kg of resveratrol could significantly increase the VH and VCR and increase the plasma D-lactic acid level and diamine oxidase activity of piglets under the challenge of diquat dibromide [35]. However, the effect of porous zinc oxide on intestinal morphology was not as good as that of high-dose zinc oxide in our study, which may be due to the low dosage of porous zinc oxide in the experiment and the relatively reduced contact with the intestinal surface after the mixing of chyme. In this study, compared with the NC group, the VH of the duodenum supplemented with plant polyphenols showed an increasing trend, indicating that plant polyphenols have the potential to improve the intestinal morphology and nutrient absorption efficiency of weaned piglets. At the same time, the VCR of the duodenum, jejunum, and ileum of the PP group was gradually increased to 1.68, 1.92, and 2.24, indicating that plant polyphenols may be mainly digested and absorbed in the back part of the intestine and play their functions. In addition, the VCR of the jejunum in the compound addition group was higher than that in the NC group, which was consistent with that in the PC group, indicating that porous zinc oxide and plant polyphenols had a certain synergistic effect on improving intestinal morphology and had the best effect on the jejunum. The composition and structure of the intestinal microbial community and its metabolites affect the body health status, and the distribution of the microbial community is affected by diet, age, and physiological state. Zinc oxide does not play a direct bactericidal role, but prevents bacterial adhesion and internalization [32,36], while inhibiting the formation of biofilm, which is a non-specific inhibitory effect [37]. Thus, it can reduce the damage of pathogenic bacteria to intestinal mucosa, improve intestinal immunity, and effectively improve the occurrence of diarrhea in weaned piglets. It was found that the ratio of Bacteroidetes to Firmicutes and the abundance of the *Prevotella* genus in the feces of piglets with diarrhea was significantly lower than the control group and anti-diarrhea group piglets, while the abundance of the *Escherichia coli* Shigella genus increased [38]. In this study, the supplementation of high-dose zinc oxide and porous zinc oxide in the diet decreased the relative abundance of Firmicutes and increased the relative abundance of Bacteroidetes, and then decreased the ratio of Firmicutes and Bacteroidetes, which might be the reason for improving the intestinal health of the weaned piglets. The relative abundance of *Lactobacillus* and *Prevotella* in the PC and PZ groups were higher than that in the NC group, indicating that the dietary addition of high-dose zinc oxide or porous zinc oxide was more conducive to the reproduction and growth of beneficial bacteria in the intestinal tract of weaned piglets. Dietary zinc oxide supplementation could significantly reduce the number of OTUs and the Chao1 index of microorganisms in the cecum contents of weaned piglets, while increasing the relative abundance of *Prevotella*, *Blastomycetes*, *Streptococcus*, and *Macrosphaera* [39]. Feeding high-dose zinc oxide was reported to increase the Chao1 index of ileal contents in piglets, while decreasing the relative abundance of Streptococcus, Blautia, Bacteroides, and Roseburia [40]. We observed that the relative abundances of *Lactobacillus*, *Prevotella*, *Blautia*, and *Sarcina* in diets supplemented with plant polyphenols were lower than those in the NC group, indicating that plant polyphenols decreased the abundance of harmful bacteria and the relative abundance of beneficial bacteria. Plant polyphenols may play a potential probiotic role by regulating the structure of intestinal flora. Dietary supplementation with 0.5% Red osier dogwood polyphenols increased the number of unique OTUs in the ileum contents, the α diversity was significantly increased, and the relative abundance of the Lactobacillus family was increased from 5.92% to 35.09% [41]. Dietary hydroxytyrosol can increase the relative abundance of beneficial bacteria and decrease the relative abundance of harmful bacteria, as well as increasing plasma steroid hormones such as testosterone and antioxidant molecules [42]. Dietary chlorogenic acid supplementation significantly increased cecal α diversity, significantly increased the Firmicutes and Bacteroidetes relative abundance, and significantly decreased the Proteobacteria relative abundance [43]. Similar with other parameters in our study, we observed the synergistic effect of porous zinc oxide and plant polyphenols in the cecal microbiota of weaned piglets. The relative abundance of *Lactobacillus* and *Blautia* in the PZ + PP group was lower than that in the PZ group but higher than that in the PP group; the relative abundance of *Prevotella* in the PZ + PP group was lower than that in the PZ and PP groups; and the relative abundance of *Sarcina* in the PZ + PP group was higher than that in NC group. These results indicate that the addition of porous zinc oxide can compensate for the decrease in plant polyphenols in the abundance of beneficial bacteria. Thus, the use of porous zinc oxide alone or combined with plant polyphenols in the diet is more conducive to optimizing the intestinal microbial community structure and maintaining healthy microflora distribution.

## 5. Conclusions

The addition of porous zinc oxide in the diet modulated the reproduction of beneficial bacteria in the intestinal tract of weaned piglets, and dietary supplementation with plant polyphenols improved the intestinal morphology of weaned piglets. Compared with the PZ and PP supplementation, the combination of PZ and PP showed better intestinal health. Therefore, porous zinc oxide and plant polyphenols have a synergistic effect on improving the intestinal health of weaned piglets and can be used as a substitute for high-dose zinc oxide.

## Figures and Tables

**Table 1:** Composition and analyzed nutrient composition of experimental diets (as-fed basis).

Item	Pre-Starter (0–14 Days)	Starter (14–42 Days)
Ingredients, %		
Extruded corn	48.40	63.58
Soybean meal, 46%	14.60	17.50
Extruded soybean	11.50	5.00
Fish meal, 68%	5.00	3.00
Whey power	15.00	5.00
Soybean oil	1.00	1.20
CaH_2_PO_4_	0.40	0.60
Limestone	0.80	1.00
NaCl	0.30	0.30
Choline chloride (60%)	0.05	0.05
Lysine	1.20	1.10
Methionine	0.09	0.09
Threonine	0.27	0.25
Tyrosine	0.02	0.01
Phytase	0.02	0.02
Organic acids	0.20	0.20
Butyric acid	0.15	0.10
Premix ^1^	1.00	1.00
Nutrient levels		
CP, %	19.50	18.00
Ca, %	0.80	0.70
Total P, %	0.65	0.60
ME, MJ//kg	14.47	13.70
Lysine, %	1.30	1.15
Methionine, %	0.38	0.34
Threonine, %	0.76	0.68
Tyrosine, %	0.21	0.18

^1^ Premix supplied per kg of diet: vitamin A, 35.2 mg; vitamin D_3_, 7.68 mg; vitamin E, 128 mg; vitamin K_3_, 8.16 mg; vitamin B_1_, 4 mg; vitamin B_2_, 12 mg; vitamin B_6_, 8.32 mg; vitamin B_12_, 4.8 mg; niacin, 38.4 mg; calcium pantothenate, 25 mg; folic acid, 1.68 mg; biotin, 0.16 mg; iron (FeSO_4_·H_2_O), 171 mg; manganese (MnSO_4_·H_2_O), 42.31 mg; zinc (ZnSO_4_·H_2_O), 110 mg; copper (CuSO_4_·5H_2_O), 125 mg; selenium (Na_2_SeO_3_), 0.19 mg; cobalt (CoCl_2_), 0.19 mg; iodine (Ca(IO_3_)_2_), 0.54 mg.

**Table 2:** Effect of porous zinc oxide (PZ), plant polyphenols (PP), or their combination (PZ + PP) supplementation on growth performance and diarrhea incidence of weaned piglets compared to a basal diet and the basal diet containing ZnO.

Item	NC	PC	PZ	PP	PZ + PP	SEM	*p* Value
BW, kg							
d 0	7.19	7.19	7.19	7.19	7.19	0.55	1.000
d 14	9.67	10.04	9.67	9.59	9.78	0.76	0.351
d 28	15.54	15.93	15.73	15.38	16.24	1.15	0.643
d 42	23.55	24.03	23.47	22.90	24.84	1.50	0.496
ADG, g/d							
d 0 to 14	177	204	177	168	184	15	0.296
d 14 to 28	419	420	433	414	462	33	0.672
d 28 to 42	572	578	553	538	614	34	0.535
d 0 to 42	366	383	379	352	399	25	0.483
ADFI, g/d							
d 0 to 14	298	306	267	288	308	24	0.356
d 14 to 28	643 ^xy^	603 ^xy^	569 ^y^	681 ^x^	627 ^xy^	47	0.099
d 28 to 42	928	915	911	967	959	53	0.779
d 0 to 42	623	608	583	645	631	41	0.453
FCR							
d 0 to 14	1.68	1.50	1.52	1.74	1.71	0.08	0.280
d 14 to 28	1.54	1.45	1.32	1.71	1.36	0.09	0.113
d 28 to 42	1.63 ^ab^	1.58 ^ab^	1.66 ^ab^	1.83 ^a^	1.56 ^b^	0.06	0.049
d 0 to 42	1.70 ^ab^	1.59 ^b^	1.54 ^b^	1.85 ^a^	1.58 ^b^	0.05	0.009
Diarrhea incidence, %							
d 0 to 14	9.07	6.19	8.33	10.75	7.86	-	0.204

^a, b^ Means listed in the same row with different superscripts are significantly different (*p ≤ 0.05*); ^x, y^ Means listed in the same row with different superscripts tend to be different (0.05 < *p* ≤ 0.10). NC = basal diet; PC = basal diet with 2000 mg/kg of zinc oxide in the pre-starter phase; PZ = basal diet with 500 mg/kg of porous zinc oxide in the pre-starter phase; PP = basal diet with 1500 mg/kg of plant polyphenols in the pre-starter phase and 1000 mg/kg of plant polyphenols in the starter phase; PZ + PP = basal diet with 500 mg/kg of porous zinc oxide and 1500 mg/kg of plant polyphenols in the pre-starter phase and 1000 mg/kg of plant polyphenols in the starter phase.

**Table 3:** Effect of porous zinc oxide (PZ), plant polyphenols (PP), or their combination (PZ + PP) supplementation on intestinal morphology of weaned piglets compared to a basal diet and the basal diet containing ZnO.

Item	NC	PC	PZ	PP	PZ + PP	SEM	*p* Value
Duodenum							
Villus height, µm	334 ^y^	418 ^xy^	405 ^xy^	454 ^x^	438 ^xy^	23	0.077
Crypt depth, µm	221	263	221	276	247	21	0.400
V:C ratio	1.54	1.61	1.95	1.68	1.90	0.12	0.195
Jejunum							
Villus height, µm	317 ^y^	399 ^x^	395 ^xy^	398 ^xy^	404 ^x^	18	0.055
Crypt depth, µm	199	182	246	213	201	13	0.105
V:C ratio	1.65 ^b^	2.27 ^a^	1.65 ^b^	1.92 ^ab^	2.14 ^a^	0.07	0.002
Ileum							
Villus height, µm	294	315	335	302	347	14	0.150
Crypt depth, µm	187	130	182	142	169	18	0.187
V:C ratio	1.67 ^y^	2.52 ^x^	1.95 ^xy^	2.24 ^xy^	2.13 ^xy^	0.16	0.052

^a, b^ Means listed in the same row with different superscripts are significantly different (*p* ≤ 0.05); ^x, y^ Means listed in the same row with different superscripts tend to be different (0.05 < *p* ≤ 0.10). NC = basal diet; PC = basal diet with 2000 mg/kg of zinc oxide in the pre-starter phase; PZ = basal diet with 500 mg/kg of porous zinc oxide in the pre-starter phase; PP = basal diet with 1500 mg/kg of plant polyphenols in the pre-starter phase and 1000 mg/kg of plant polyphenols in the starter phase; PZ + PP = basal diet with 500 mg/kg of porous zinc oxide and 1500 mg/kg of plant polyphenols in the pre-starter phase and 1000 mg/kg of plant polyphenols in the starter phase.

**Table 4:** Effect of porous zinc oxide (PZ), plant polyphenols (PP), or their combination (PZ + PP) supplementation on α diversity in the cecal contents of weaned piglets compared to a basal diet and the basal diet containing ZnO.

Item	NC	PC	PZ	PP	PZ + PP	SEM	*p* Value
ACE	436	418	470	473	450	69	0.984
Chao	441	424	481	496	468	68	0.960
Shannon	0.06	0.05	0.05	0.05	0.08	0.25	0.909
Simpson	3.80	3.84	3.99	4.05	3.71	0.02	0.727

NC = basal diet; PC = basal diet with 2000 mg/kg of zinc oxide in the pre-starter phase; PZ = basal diet with 500 mg/kg of porous zinc oxide in the pre-starter phase; PP = basal diet with 1500 mg/kg of plant polyphenols in the pre-starter phase and 1000 mg/kg of plant polyphenols in the starter phase; PZ + PP = basal diet with 500 mg/kg of porous zinc oxide and 1500 mg/kg of plant polyphenols in the pre-starter phase and 1000 mg/kg of plant polyphenols in the starter phase.

## Data Availability

The data presented in this study are available on request from the corresponding authors.
